# Implementation of a sagittal T2-weighted DIXON turbo spin-echo sequence may shorten MRI acquisitions in the emergency setting of suspected spinal bleeding

**DOI:** 10.1186/s41747-021-00213-5

**Published:** 2021-05-12

**Authors:** Nico Sollmann, Charlotte Rüther, Simon Schön, Claus Zimmer, Thomas Baum, Jan S. Kirschke

**Affiliations:** 1 Department of Diagnostic and Interventional Neuroradiology, School of Medicine, Klinikum rechts der Isar, Technical University of Munich, Ismaninger Str. 22, 81675 Munich, Germany; 2 TUM-Neuroimaging Center, Klinikum rechts der Isar, Technical University of Munich, Munich, Germany; 3 Department of Diagnostic and Interventional Radiology, University Hospital Ulm, Albert-Einstein-Allee 23, 89081 Ulm, Germany

**Keywords:** Clinical protocols, DIXON, Emergency service (hospital), Magnetic resonance imaging, Spine

## Abstract

**Background:**

Magnetic resonance imaging (MRI) is the modality of choice for evaluating soft tissue damage along the spine in the emergency setting, yet access and fast protocol availability are limited. We assessed the performance of a sagittal T2-weighted DIXON turbo spin-echo sequence and investigated whether additional standard sagittal T1-weighted sequences are necessary in suspected spinal fluid collections/bleedings.

**Methods:**

Seventy-four patients aged 62.9 ± 19.3 years (mean ± standard deviation) with MRI including a sagittal T2-weighted DIXON sequence and a T1-weighted sequence were retrospectively included. Thirty-four patients (45.9%) showed a spinal fluid collection/bleeding. Two layouts (layout 1: fat-only and water-only and in-phase images of the DIXON sequence and T1-weighted images; layout 2: fat-only and water-only and in-phase images of the DIXON sequence) were evaluated by three readers (R1, R2, and R3) concerning presence of spinal fluid collections/bleedings and diagnostic confidence from 1 (very low confidence) to 5 (very high confidence). *χ*^2^ and *κ* statistics were used.

**Results:**

There was no difference in detecting spinal fluid collections/bleedings between the layouts (R1 and R2 detected all, R3 missed one spinal fluid collection/bleeding in the same patient in both layouts). Confidence was high (layout 1, R1 4.26 ± 0.81, R2 4.28 ± 0.81, R3 4.32 ± 0.79; layout 2, R1 3.93 ± 0.70, R2 4.09 ± 0.86, R3 3.97 ± 0.73), with higher inter-reader agreement for layout 1 (*κ* 0.691–0.780) than for layout 2 (*κ* 0.441–0.674).

**Conclusions:**

A sagittal T2-weighted DIXON sequence provides diagnostic performance similar to a protocol including standard T1-weighted sequences.

## Key points

- Magnetic resonance imaging (MRI) is key for evaluation of soft tissue damage at the spine in the emergency setting.
- A sagittal T2-weighted DIXON turbo spin-echo (TSE) sequence may be enough for this purpose and could shorten MRI acquisitions.
- There was no difference in detection of spinal fluid collections/bleedings between layouts using DIXON with or without additional T1-weighted images.
- A sagittal T2-weighted DIXON TSE sequence provides diagnostic performance similar to a protocol including standard T1-weighted sequences.

## Background

Imaging plays a key role in the emergency setting of patients with suspected spinal fluid collections/bleedings, which are observed primarily in the context of trauma, but also after spinal surgical procedures or, in rare cases, spontaneously. Compressive effects due to the space-occupying character of spinal fluid collections/bleedings can entail severe neurological symptoms when the spinal cord or exiting spinal nerves are affected. Rapid clinical and radiological evaluations are important to decide on the treatment strategy and to optimise outcome.

Due to wide availability and speed of examination, computed tomography (CT) is regarded the initial modality of imaging [1, 2]. In most scenarios, non-contrast CT with multi-planar reconstructions is routine and considered gold standard for identifying fractures along the spine with a reported sensitivity of 94% to 100% [2–4]. In selected cases, CT myelography may be considered to specifically detect traumatic spinal canal narrowing due to disco-ligamentous injury or epidural haematoma, or to evaluate preganglionic nerve root avulsions [2]. Yet, both conventional CT and CT myelography are considered inferior to magnetic resonance imaging (MRI) in assessing most trauma consequences except for mere vertebral fractures with or without spinal compression [2, 5].

Specifically, MRI is indicated when neurological involvement is present and/or to assess soft tissue damage in case of trauma, such as disco-ligamentous injury or epidural space compromise [1, 2, 6–8]. Although there is no definite consensus on the exact MRI protocol to be used in such patients, a multi-sequence approach consisting of at least sagittal T1- and T2-weighted spin echo sequences, sagittal short tau inversion recovery (STIR) sequences, and axial T2-weighted sequences tailored to the levels of pathology seems to be routine [8–10]. Further sequences may be added depending on individual needs, such as T2*-weighted gradient-recalled echo or diffusion-weighted sequences [8–10].

However, such multi-sequence scanning protocols for MRI can take a considerable amount of time, which is a crucial issue in an emergency patient. Specifically, long acquisitions may harbor a risk for unfavourable outcome due to delayed initiation of therapy related to long scanning time, and patients with spinal fluid collections/bleedings often suffer from pain and/or neurological deficits that may cause increasing discomfort and moving artifacts in imaging data with long protocols. Hence, protocol optimisation for emergency MRI of the spine are welcomed, with the aim of facilitating acquisitions with reduced scanning time while not restricting diagnostic value of examinations.

In this regard, the DIXON technique combined with turbo spin-echo (TSE) imaging may be an alternative to consecutive acquisitions of sagittal T1- and T2-weighted and STIR sequences. The DIXON technique represents a chemical shift imaging method for water and fat separation in MRI, acquiring separate images with a modified spin echo pulse sequence [10–12]. Within a one-sequence acquisition, a T2-weighted DIXON sequence can deliver four image sets that differ in their contrasts, including so-called fat-only images (comparable to standard T1-weighted sequences), water-only images (comparable to STIR sequences), in-phase images (comparable to standard T2-weighted sequences), and out-of-phase images [10, 11, 13, 14]. Specifically, acquiring sagittal T2-weighted DIXON sequences exclusively—instead of acquiring additional T1-weighted sequences—could save scan time and limit the duration of time inside the scanner for emergency patients, decreases costs for the MRI session, and could limit motion artifacts as a possible result of long acquisition times. However, despite these benefits, a systematic evaluation of the utility of a T2-weighted DIXON sequence in the emergency patient with suspected spinal fluid collections/bleedings for protocol optimisation is lacking to date.

Against this background, this study aims to investigate the combination of fat-only, water-only, and in-phase images as derived from a single sagittal T2-weighted DIXON sequence in comparison to images of the sagittal T2-weighted DIXON sequence plus a dedicated T1-weighted sequence. We hypothesised that the sagittal T2-weighted DIXON sequence is sufficient, thus probably making additional sagittal T1-weighted imaging obsolete.

## Methods

### Study design and ethics

This study is a retrospective, cross-sectional observational study. Under the inclusion and exclusion criteria, a consecutive series of patients at a single institution was enrolled. The study was approved by the local institutional review board and was conducted in accordance with the Declaration of Helsinki. The need for written informed consent was waived by the institutional review board due to the retrospective design of the study.

### Study inclusion

Eligible patients were retrospectively identified in our hospital’s Picture Archiving and Communication System (PACS), with the interval of study inclusion ranging from November 2018 to March 2019. The following inclusion criteria were considered: (1) MRI performed for clinically suspected spinal fluid collections/bleedings and/or spinal injury and/or unclear neurological deficits, (2) acquisition of at least a sagittal T2-weighted DIXON sequence and a conventional sagittal T1-weighted sequence at 3 Tesla, and (3) age of above 18 years. Patients were excluded when the following criteria were met: (1) motion artifacts in imaging data, (2) severe artifacts due to implants at the level of (suspected) pathology, resulting in non-diagnostic image quality, (3) severe scoliosis, and (4) congenital disorders with severe structural aberrations of the spine and/or spinal cord (*e.g.,* spina bifida or tethered cord).

Overall, 74 patients fulfilled the inclusion criteria (age 62.9 ± 19.3 years, mean ± standard deviation (SD); range 20.5-92.2 years, 55.4% females). Imaging in these patients was performed for clinically indicated reasons only, and included patients without previous surgery (*e.g.,* initial emergency imaging for acute trauma) as well as patients with a history of previous surgery (*e.g.,* postoperative imaging after spinal instrumentation to rule out haematoma as a surgical complication). In patients with spinal fluid collections/bleedings, pathology was located either extraspinal (prevertebral or in dorsal soft tissue), intraspinal (epidural, intradural extramedullary, or intramedullary), or constituted a combination of extra- and intraspinal manifestation. Patients were referred to our neuroradiological department by neurosurgeons, neurologists, or trauma and orthopaedic surgeons.

### Magnetic resonance imaging

Imaging of the spine was performed with a 3-T scanner (Achieva dStream or Ingenia; Philips Healthcare, Best, The Netherlands) in combination with body coils. Scanning was conducted in supine position, with sequences being obtained subsequent to manual planning within an initially acquired survey scan. The field of view (FOV) covered the cervical, thoracic, or lumbar spine or a combination of those, depending on clinical indication. The following sequences were acquired in all included patients:

- Sagittal T2-weighted DIXON TSE sequence (repetition time [TR]/echo time [TE] = 2500/100 ms, acquisition voxel size 0.70 × 0.98 × 3.00 mm, FOV 180 × 275 × 49 mm, acquisition time 3 min 25 s)
- Non-contrast-enhanced sagittal T1-weighted sequence (TR/TE = 600/8 ms, acquisition voxel size 0.80 × 1.00 × 3.00 mm, FOV 180 × 275 × 49 mm, acquisition time 3 min 3 s).

All acquisitions were accelerated using Compressed SENSE by default. Depending on diagnostic needs, further sequences (*e.g.,* axial T2-weighted sequences at the level of suspected pathology) were added, but not taken into account for this study. Prior to implementation of the sagittal T2-weighted DIXON sequences at our department, conventional sagittal T2-weighted TSE and STIR sequences were acquired in addition to the sagittal T1-weighted sequence, which had acquisition durations of 3 min 54 s (sagittal T2-weighted TSE sequence) and 4 min 44 s (sagittal STIR sequence), respectively.

### Image analysis

Qualitative image evaluation was performed by three independent readers with different expertise: reader 1 (R1), consultant in neuroradiology with 9 years of experience; reader 2 (R2), fellow in neuroradiology with 7 years of experience; reader 3 (R3), third-year resident in neuroradiology. The three readers used the PACS viewer (IDS7; Sectra AB, Linköping, Sweden). They were blinded to the individual clinical indications for MRI, the patients’ clinical symptoms, and the radiological reports created during clinical routine. Furthermore, they were strictly blinded to the evaluations of each other.

Each reader evaluated two different image layouts, with an interval of at least 8 weeks between assessments of both layouts. During evaluation of each layout, the order of patient cases was subject to randomisation. Readers were advised to determine whether a spinal fluid collection/bleeding was present or not considering the entire FOV, providing a binary decision: 1, presence of a spinal fluid collection; 0, no detectable spinal fluid collection. Furthermore, diagnostic confidence was determined on a 5-point Likert scale: 1, very low diagnostic confidence; 2, low diagnostic confidence; 3, intermediate diagnostic confidence; 4, high diagnostic confidence; 5, very high diagnostic confidence.

The two image layouts were arranged as defined in the following:

- Layout 1, fat-only images AND water-only images AND in-phase images as derived from the sagittal T2-weighted DIXON sequence AND sagittal T1-weighted sequence
- Layout 2, fat-only images AND water-only images AND in-phase images as derived from the sagittal T2-weighted DIXON sequence

A senior consultant in neuroradiology with 10 years of experience performed additional image evaluation. The minimum dataset for reference standard reading by the consultant comprised the sagittal T2-weighted DIXON sequence with all four image sets (fat-only images, water-only images, in-phase images, and out-of-phase images), the sagittal T1-weighted sequence, as well as all axial T1- and/or T2-weighted sequences. In addition, the reference standard evaluation included all other imaging data available in the PACS per patient (*e.g.,* follow-up MRI or CT imaging studies), as well as the entire medical history including access to the medical charts, surgical reports, and radiological reports. This additional image evaluation was conducted for two purposes: (1) to determine absence/presence of spinal fluid collections/bleedings on MRI of the spine; (2) to determine the signal characteristics of the spinal fluid collections/bleedings, if any (isointense, hypo-, or hyperintense in the image sets derived from the sagittal T2-weighted DIXON sequence and in the sagittal T1-weighted sequence).

### Statistical analysis

GraphPad Prism (version 6.0; GraphPad Software Inc., San Diego, CA, USA) and SPSS (version 26.0; IBM SPSS Statistics for Windows, IBM Corp., Armonk, NY, USA) were used for statistical data analysis. The level of statistical significance was set at *p* < 0.05.

Descriptive statistics including absolute and relative frequencies, mean, SD, and ranges were calculated. *χ*^2^ tests were conducted for the distribution of presence or absence of detected spinal fluid collections/bleedings between the three readers, which were achieved separately for the two image layouts. Scores assigned for diagnostic confidence per layout were compared between the three readers using quadratic weighted Cohen’s *κ*. Additionally, κ was also computed to evaluate intra-reader agreement between scoring assigned for the two image layouts. The following scale for interpretation of κ was considered: 0.00, no agreement; 0.00–0.20, slight agreement; 0.21–0.40, fair agreement; 0.41–0.60, moderate agreement; 0.61–0.80, substantial agreement; 0.81–1, almost perfect agreement.

## Results

### Cohort characteristics

In the 74 included patients, emergency MRI of the spine was clinically indicated due to previous trauma in 39 patients (52.7%) and in the context of postoperative evaluation (after spinal surgical procedures for tumour resection or due to degenerative spine conditions) in 18 patients (24.3%). According to the reference standard reading, 34 patients (45.9%) showed spinal fluid collections/bleedings.

Among these 34 patients, 17 patients (50.0%) showed a combination of intraspinal and dorsal extraspinal, and 4 patients (11.8%) showed prevertebral and dorsal extraspinal fluid collections/bleedings. Isolated prevertebral or isolated dorsal extraspinal fluid collections/bleedings were detected in 4 patients each (11.8% each), and isolated intraspinal pathology was seen in 5 patients (14.6%). In the 22 patients with intraspinal fluid collections/bleedings, pathology was found epidural in 16 patients (72.7%), intradural extramedullary in 5 patients (22.7%), and intramedullary in the remaining patient (4.6%).

Holospinal scanning was performed in 28 patients (37.8%), while only the cervical, thoracic, or lumbar spine was investigated in 10 (13.5%), 5 (6.8%), and 15 patients (20.3%), respectively. Six patients (8.1%) underwent MRI of a combination of the cervical and thoracic spine, the remaining 10 patients (13.5%) underwent scanning of both the thoracic and lumbar spine in one session.

### Diagnostic performance

**Fig. 1:**
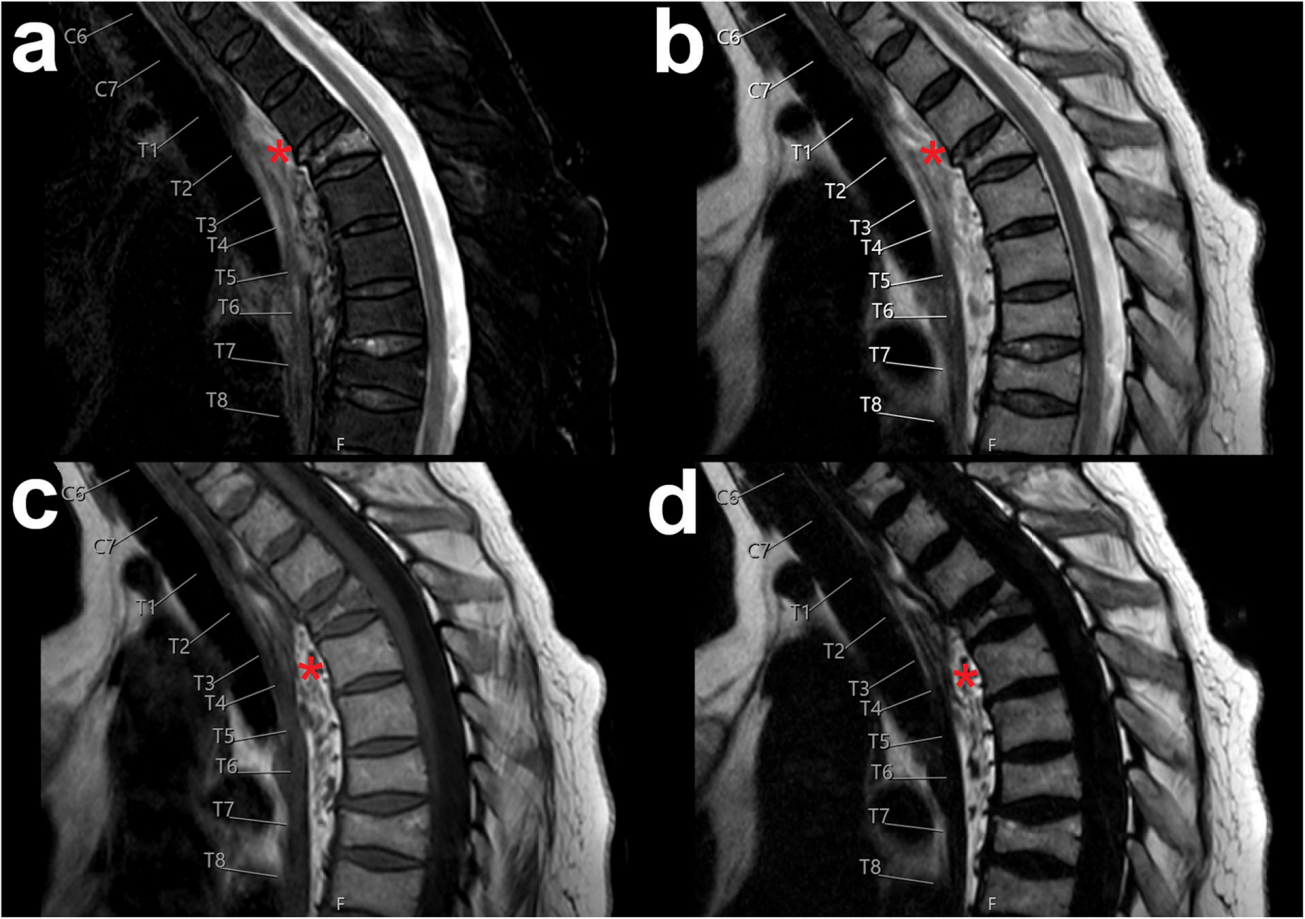
Traumatic thoracic prevertebral haematoma. This figure shows the thoracic spine of a 62-year-old female patient using water-only images (**a**), in-phase images (**b**), and fat-only images (**d**) of the sagittal T2-weighted DIXON sequence, together with a sagittal T1-weighted sequence (**c**). The patient had suffered a traffic accident and showed a thoracic prevertebral haematoma spanning from T1 to T8 (red asterisks) combined with an acute vertebral fracture (T3)

**Fig. 2:**
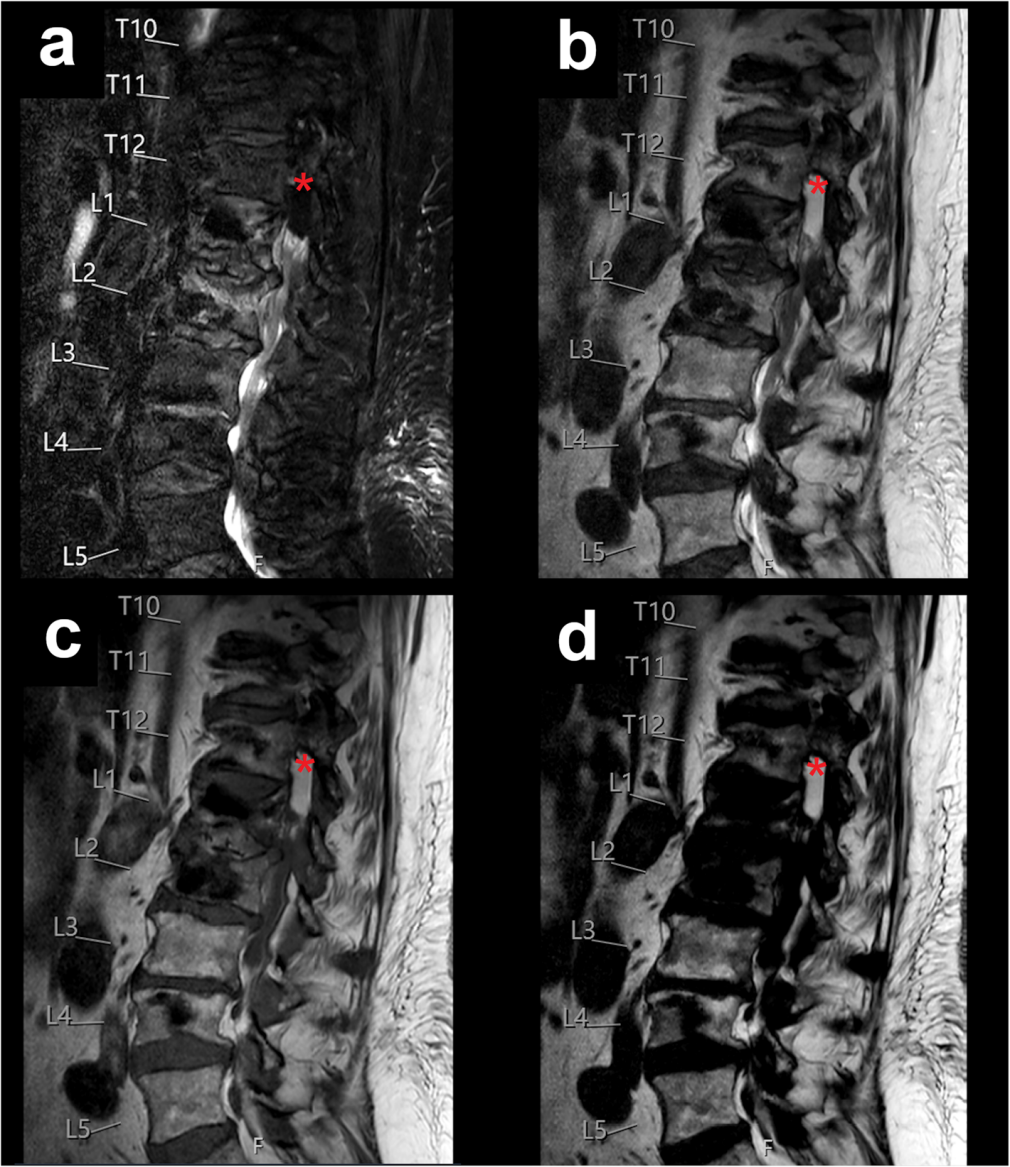
Postoperative lumbar haematoma. This figure shows the lumbar spine of an 80-year-old female patient using water-only images (**a**), in-phase images (**b**), and fat-only images (**d**) of the sagittal T2-weighted DIXON sequence, together with a sagittal T1-weighted sequence (**c**). The patient had undergone decompressive lumbar hemilaminectomy and presented with a small lateralised postoperative haematoma of the intradural extramedullary space between T12 and L1 (red asterisks)

**Fig. 3:**
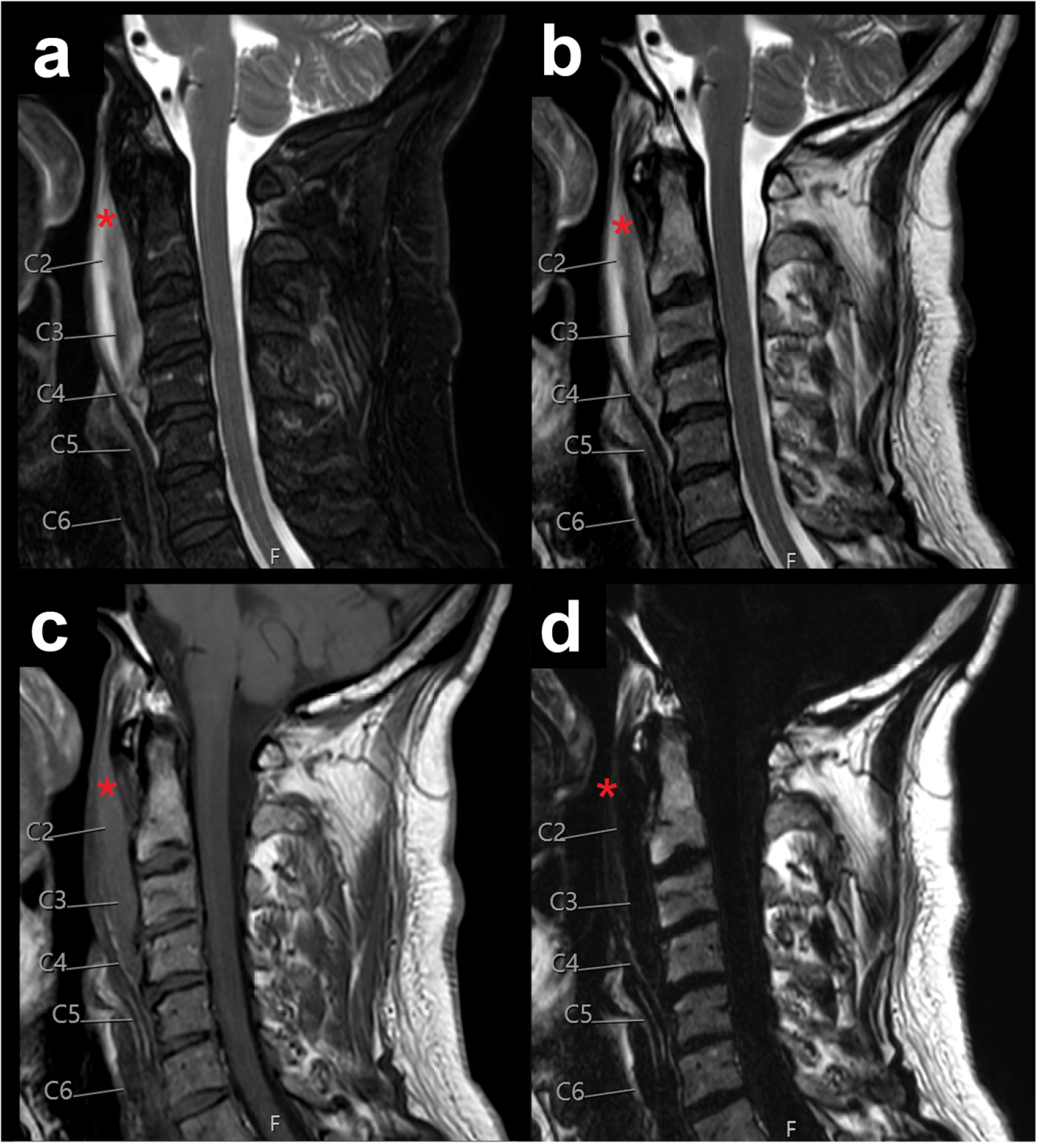
Traumatic cervical prevertebral haematoma. This figure shows the cervical spine of a 55-year-old male patient using water-only images (**a**), in-phase images (**b**), and fat-only images (**d**) of the sagittal T2-weighted DIXON sequence, together with a sagittal T1-weighted sequence (**c**). The patient had suffered a traffic accident and shows a cervical prevertebral haematoma spanning from C1 to C5 (red asterisks)

**Fig. 4:**
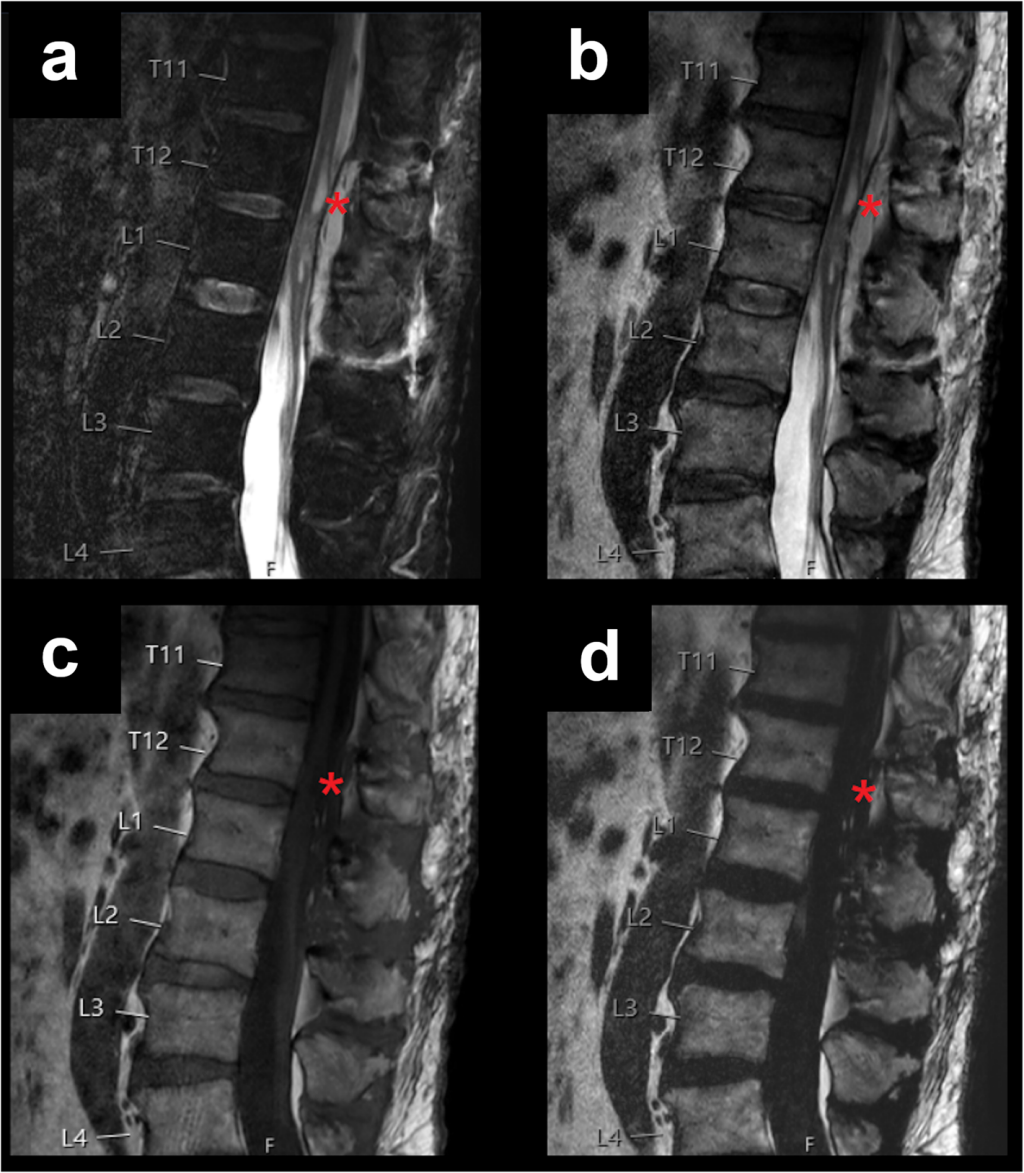
Postoperative thoracolumbar haematoma. This figure shows the lower thoracic and lumbar spine of a 69-year-old male patient using water-only images (**a**), in-phase images (**b**), and fat-only images (**d**) of the sagittal T2-weighted DIXON sequence, together with a sagittal T1-weighted sequence (**c**). The patient had undergone decompressive lumbar surgery and presented with a postoperative epidural haematoma between T12 and L2 (red asterisks)

**Fig. 5:**
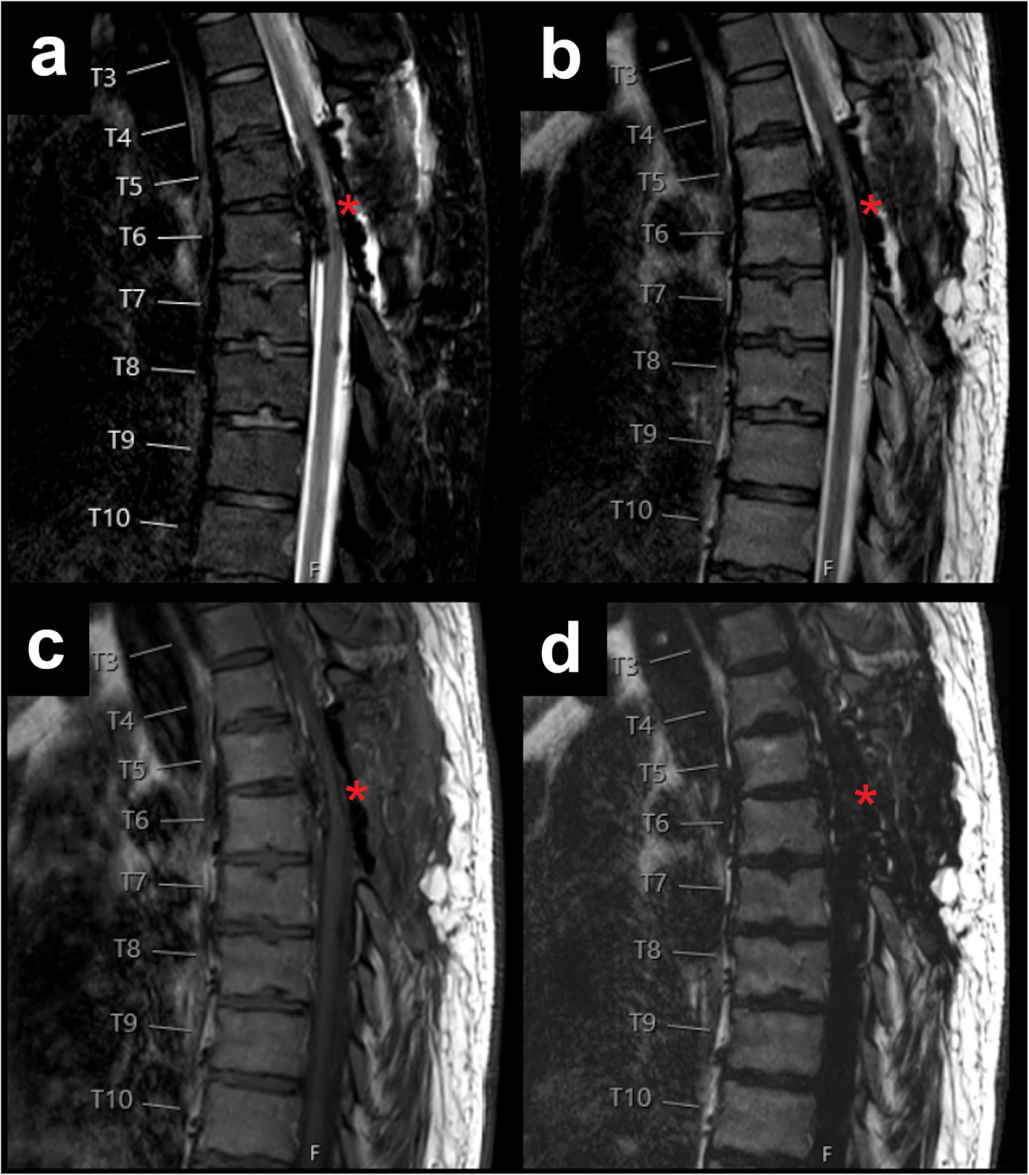
Postoperative thoracic haematoma. This figure shows the thoracic spine of a 39-year-old female patient using water-only images (**a**), in-phase images (**b**), and fat-only images (**d**) of the sagittal T2-weighted DIXON sequence, together with a sagittal T1-weighted sequence (**c**). The patient had undergone decompressive thoracic surgery and presented with a postoperative intradural extramedullary haematoma between T4 and T7 (red asterisks)

Exemplary patient cases with spinal fluid collections/bleedings are shown in Figs. 1, 2, 3, 4, and 5, comprising representative sagittal images of the fat-only images, water-only images, and in-phase images of the T2-weighted DIXON sequence as well as sagittal images of the T1-weighted sequence.

R1 and R2 detected all spinal fluid collections/bleedings in the affected 34 patients (100%) during separate evaluations of both image layouts, respectively. R3 missed one spinal fluid collection/bleeding, thus only detecting spinal fluid collections/bleedings in 33 patients (97.1%). This finding was missed in the same patient during assessment of both image layouts. None of the readers erroneously assigned a spinal fluid collection/bleeding to a patient not showing such pathology according to reference standard reading.

**Fig. 6:**
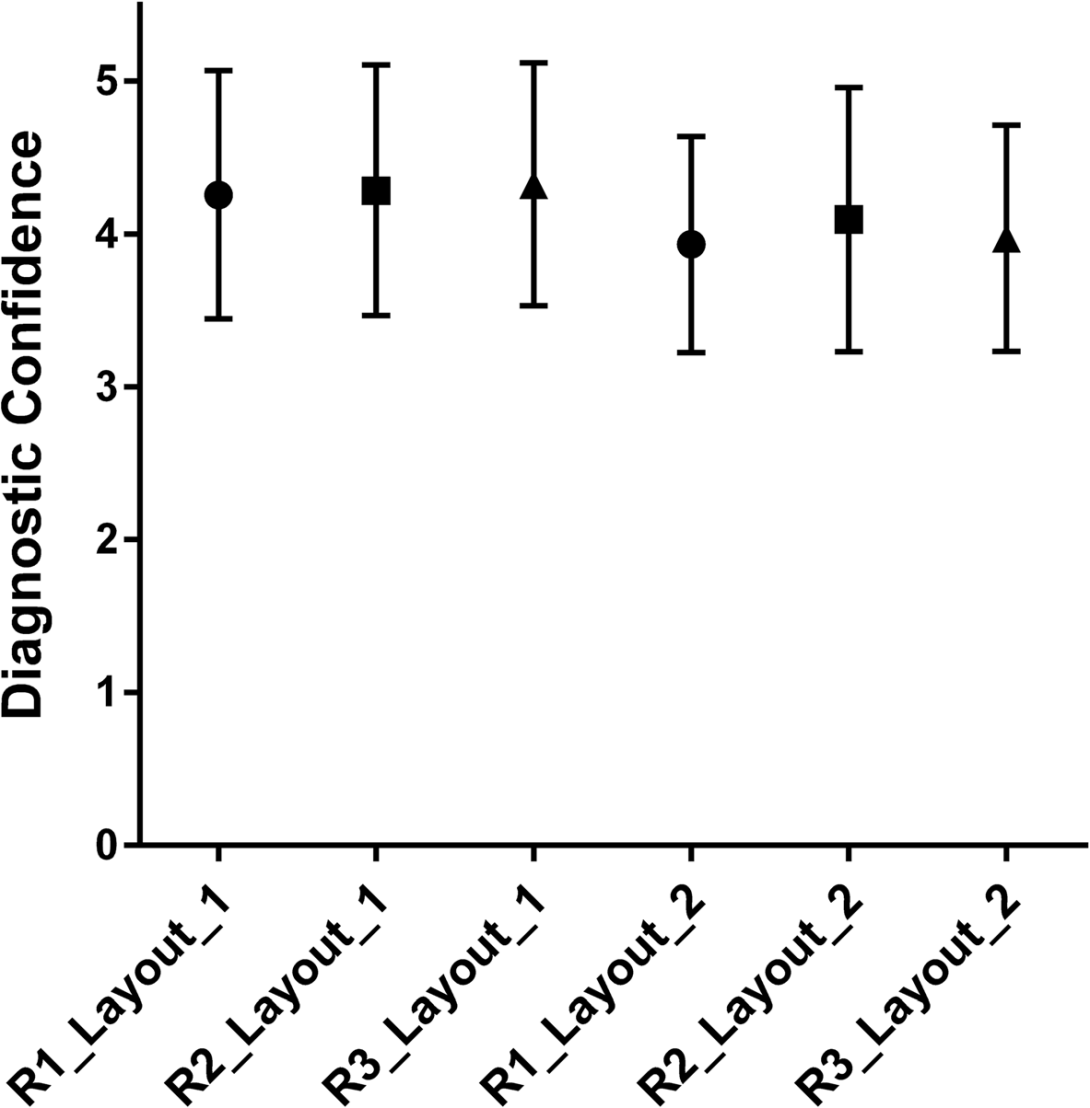
Diagnostic confidence. This graph plots the mean scores ± standard deviation for the diagnostic confidence as assigned by the three readers (R1, R2, and R3) for the two image layouts: layout 1, fat-only images AND water-only images AND in-phase images as derived from the sagittal T2-weighted DIXON sequence AND sagittal T1-weighted sequence; layout 2, fat-only images AND water-only images AND in-phase images as derived from the sagittal T2-weighted DIXON sequence

On average, diagnostic confidence was high for evaluation of both image layouts according to all readers, yet scores assigned during reading of the second image layout were slightly lower: layout 1, R1 4.26 ± 0.81, R2 4.28 ± 0.81, R3 4.32 ± 0.79; layout 2, R1 3.93 ± 0.70, R2 4.09 ± 0.86, R3: 3.97 ± 0.73 (Fig. 6). Analogously, inter-reader agreement was better for layout 1 (*κ* 0.691–0.780) than for layout 2 (*κ* 0.441–0.674) (Table 1). Intra-reader agreement was moderate to substantial when comparing scorings for the two image layouts for diagnostic confidence (*κ* 0.460–0.685).

**Table 1:** Inter-reader agreement for diagnostic confidence as measured by quadratic weighted Cohen’s *κ* for the comparison of the scorings of the three readers (R1, R2, and R3) regarding the two image layouts

	Layout 1	Layout 2
R1 *versus* R2	0.691	0.441
R1 *versus* R3	0.778	0.674
R2 *versus* R3	0.780	0.549

### Signal intensity

According to reference standard reading, the signal characteristics (isointense, hypo-, or hyperintense signal of the detected spinal fluid collections) were rated equal in the fat-only images of the sagittal T2-weighted DIXON sequence and in the conventional sagittal T1-weighted sequence. Thus, there were no discrepancies in signal characteristics between these two image datasets.

In detail, detected spinal fluid collections showed a hyperintense signal in fat-only images of the T2-weighted DIXON sequence in 2 patients, while hyperintensity was found in 27 cases for water-only, in 28 cases for in-phase, and in 2 cases for T1-weighted images. Furthermore, an isointense signal was detected in 31 cases for fat-only, in 2 cases for water-only, in 2 cases for in-phase, and in 31 cases for T1-weighted images. A hypointense signal was revealed in 1 case for fat-only, in 5 cases for water-only, in 4 cases for in-phase, and in 1 case for T1-weighted images.

## Discussion

This study investigated the diagnostic performance of a sagittal T2-weighted DIXON sequence (fat-only, water-only, and in-phase images) in comparison to imaging with additional conventional sagittal T1-weighted sequences for MRI in spinal fluid collections/bleedings. There was no difference in the number of detected spinal fluid collections/bleedings between readings of both layouts, and average diagnostic confidence was high. Furthermore, signal characteristics of detected spinal fluid collections/bleedings were similar for fat-only images of the sagittal T2-weighted DIXON sequences and conventional T1-weighted sequences.

In case that MRI is indicated in patients with suspected spinal fluid collections/bleedings, fast and reliable sequence protocols are critical. Commonly, at least sagittal T1- and T2-weighted spin echo sequences, sagittal STIR sequences, and axial T2-weighted sequences are acquired [8–10]. However, within one acquisition, a sagittal T2-weighted DIXON sequence can provide fat-only, water-only, in-phase, and out-of-phase images [10, 11, 13, 14]. Thus, images of different contrasts, generally comparable to those generated during consecutive acquisition of T1- and T2-weighted and STIR sequences, are generated. In this regard, previous work has made use of the DIXON technique for MRI of different purposes. For the detection of bone metastases along the spine, it has recently been demonstrated that fat and water images derived from a T2-weighted DIXON sequence provide diagnostic performance similar to a protocol including standard T1-weighted sequences; thus, the fat images of the T2-weighted DIXON sequence might probably replace conventional T1-weighted imaging [15, 16].

In a study among patients showing various types of vertebral lesions (including recent fractures, spondylitis, metastasis, and haemangioma), a T2-weighted DIXON sequence showed more homogeneous fat suppression and superior lesion conspicuity when compared to imaging using spectral attenuated inversion recovery (SPAIR) [17]. For the degenerative lumbar spine, previous studies were able to demonstrate that imaging with a single sagittal T2-weighted DIXON sequence could replace the combination of dedicated T1-weighted, T2-weighted, and STIR sequences, which would not be at the expense of worsened diagnostic performance [18, 19].

The findings of the present study align with previous work as they provide evidence for the high potential of a sagittal T2-weighted DIXON sequence of making additional T1-weighted sequences obsolete at the spine. Yet, this is achieved for MRI in patients with suspected spinal fluid collections/bleedings, thus indications that have not been under investigation for protocol optimisation with a T2-weighted DIXON sequence to date. Given the frequent emergency situation in such patients where the need for fast diagnostics by MRI is warranted, it seems intriguing that no comparative study including a T2-weighted DIXON sequence has been conducted so far. The fat-only images derived from a T2-weighted DIXON sequence present a similar image contrast when compared to dedicated T1-weighted sequences, which potentially qualifies this image set to replace a separately acquired non-contrast-enhanced T1-weighted sequence.

According to the evaluations of three readers, the number of spinal fluid collections/bleedings detected was the same for reading of both image layouts, and the signal characteristics of detected spinal fluid collections/bleedings were highly comparable between fat-only images and the respective conventional T1-weighted sequences according to reference standard reading. Hence, the application of a T2-weighted DIXON sequence without additional T1-weighted sequences seems justified in the clinical setting. Yet, although the number of detected spinal fluid collections/bleedings was the same for both image layouts, one could speculate that T1-hypointense pathology may be the most difficult to detect, given the impression of a pronounced signal decrease in the comparable fat-only images derived from DIXON imaging. In case of equivocal findings, supplementary axial sequences of the level of suspected pathology, as also acquired in all patients of this study but not used for image analyses of the three readers for study purposes, should be particularly taken into account for diagnosis.

Furthermore, it remains at the discretion of the radiologist in charge to continue imaging with a sagittal T1-weighted sequence if diagnosis still remains uncertain in selected cases, or in case that the application of contrast media is warranted (*e.g.,* in case that initial suspicion of a spinal fluid collection or haematoma is rejected and an incidental finding of a neoplastic mass or inflammatory disease is more likely according to the initial sequences acquired). However, in the cohort of this study, diagnostic confidence was still rated high when only relying on the image sets of the T2-weighted DIXON sequence, with restrictions in inter-reader agreement being most probably attributable to initial changeover effects when the additional and commonly used T1-weighted sequences are spared during reading of the second image layout.

The main benefit of skipping sagittal T1-weighted sequences, particularly for emergency MRI, might be the reduction in overall scanning time. For this study, sparing dedicated T1-weighted imaging would reduce scanning time in our setting by 3 min 3 s, which would add up in patients in whom more than one image stack is indicated in the context of holospinal imaging. Indeed, such holospinal scanning might be required in a considerable fraction of patients especially in a posttraumatic condition, with 37.8% of this study’s patients having undergone MRI of the whole spine. When a commonly used multi-sequence protocol consisting of sagittal T1- and T2-weighted and STIR sequences is used instead of a T2-weighted DIXON sequence, the saving of scan time even becomes more prominent, again adding up in cases where acquisitions with large spatial coverage are needed. A simultaneous effect of shortened scanning time might be reflected by better overall image quality since motion artifacts tend to occur particularly for long scanning sessions, thus when multiple sequences are acquired in a row. Hence, due to acquisition of the four image sets with different contrasts within one T2-weighted DIXON sequence, motion artifacts might be limited. Alongside with savings in scanning time, another advantage of the DIXON method is that it can generate images with higher signal-to-noise ratios when compared to STIR sequences [20–23]. In addition, DIXON imaging delivers more distinct and homogeneous fat suppression when compared to other approaches, such as imaging using spectral attenuated inversion recovery (SPAIR) [17, 24, 25].

When interpreting the results of this study, we also have to acknowledge some limitations. First, a potential recall bias may be present. However, the interval between the readings of both layouts amounted to at least 8 weeks, and randomisation of patient cases was established throughout to reduce such bias. Second, absolute numbers for savings in scanning time may be specific for the MRI scanners and distinct sequence protocols used in this study, thus likely showing variations for other scanning environments at other institutions. Hence, follow-up studies using other MRI systems and acquisition parameters for scanning are needed for the purpose of external validation of our results, which may help to further optimise imaging protocols on various MRI machines. However, comparable but variable time savings should also be achieved in other setups as sparing of a T1-weighted sequence inherently leads to reduced overall acquisition time. Third, this study did not evaluate the image sets of the DIXON sequence in direct comparison to conventionally acquired T2-weighted or STIR sequences. This is due to the retrospective study design derived from a mostly clinical emergency scenario, where the need for swift scanning is of particular importance. Yet, further prospective studies are needed, *e.g.,* during the postoperative course of patients with spinal instrumentation and hemorrhage but no emergency indication, to exploit potential superiority of water-only, fat-suppressed images of the DIXON sequence over STIR images. Fourth, it needs to be emphasised that sparing T1-weighted imaging would not work for MRI exams with a requirement for contrast administration. Thus, in case that the potential need for contrast-enhanced imaging is determined, dedicated T1-weighted sequences should still be added to the scanning protocol.

In conclusion, a sagittal T2-weighted DIXON TSE sequence provides diagnostic performance similar to a protocol including standard T1-weighted sequences in emergency MRI for detection of spinal fluid collections/bleedings. Thus, additional acquisition of dedicated sagittal T1-weighted sequences could be spared, which can considerably reduce scanning time.

## Data Availability

Data are available on reasonable request from the authors.

## Abbreviations

CT: Computed tomography
FOV: Field of view
MRI: Magnetic resonance imaging
PACS: Picture Archiving and Communication System
R1: Reader 1
R2: Reader 2
R3: Reader 3
SD: Standard deviation
STIR: Short tau inversion recovery
TSE: Turbo spin-echo
